# Ultrasound Signs in the Diagnosis and Staging of Small Bowel Obstruction

**DOI:** 10.3390/diagnostics10050277

**Published:** 2020-05-03

**Authors:** Stefania Tamburrini, Nicola Serra, Marina Lugarà, Giuseppe Mercogliano, Carlo Liguori, Gabriella Toro, Francesco Somma, Ylenia Mandato, Maria Vittoria Guerra, Giuseppe Sarti, Roberto Carbone, Pasquale Tammaro, Andrea Ferraro, Roberta Abete, Ines Marano

**Affiliations:** 1 Department of Radiology, Ospedale del Mare-ASL NA1 Centro, 80147 Naples, Italy; carlo.liguori@gmail.com (C.L.); gabriella.toro@tiscali.it (G.T.); fra1585@hotmail.com (F.S.); yleniamandato@yahoo.it (Y.M.); sartigiuseppe@gmail.com (G.S.); robcarbone@alice.it (R.C.); ines.marano@tiscali.it (I.M.); 2 Department of Molecular Medicine and Medical Biotechnology, University of Naples“Federico II”, 80131 Naples, Italy; nicola.serra@gmail.com; 3 Department of Internal Medicine, Ospedale del Mare-ASL NA1 Centro, 80147 Naples, Italy; marinalugara82@gmail.com; 4 Department of Radiology, University of Naples “Federico II”, 80131 Naples, Italy; giuse.mercogliano@gmail.com; 5 Department of Emergency Medicine, Ospedale del Mare-ASL NA1 Centro, 80147 Naples, Italy; mariavittoria79.mg@gmail.com (M.V.G.); andrea.ferraro79@gmail.com (A.F.); 6 Department of Surgery, Ospedale del Mare-ASL NA1 Centro, 80147 Naples, Italy; pasqualetammaro81@gmail.com (P.T.); abeteroberta@gmail.com (R.A.)

**Keywords:** bowel ultrasound, small bowel obstruction, emergency ultrasound

## Abstract

Ultrasound (US) is highly accurate in the diagnosis of small bowel obstruction (SBO). Because the indications for and timing of surgical intervention for SBO have changed over the past several decades, there is a widespread assumption that the majority of patients with simple SBO may be conservatively managed; in this scenario, staging SBO is crucial. This study evaluated the association between morphological and functional US signs in the diagnosis and staging (simple, decompensated and complicated), and the associations and prevalence of US signs correlated with clinical or surgical outcome. The US signs were divided into diagnostic (dilated bowel loops and altered kinesis) and staging criteria (extraluminal free fluid, parietal and villi alterations). We performed a retrospective, single-center cohort, observational study examining the prevalence of morphologic and functional US signs in the staging of simple, decompensated and complicated SBO. The most significant US signs were dilated bowel loops (100%), hypokinesis (90.46%), thickened walls (82.54%) and free fluid (74.60%). By linear regression, free fluid was positively correlated to US staging in both univariate and multivariate analysis; that is, the more advanced the stage of SBO, the more probable the presence of free fluid between the bowel loops. In univariate analysis only, we found a positive correlation between US staging/thickened walls and the prominence of valvulae conniventes. Additionally, the multivariate analysis indicated that parietal stratification and bowel jump kinesis were negative predictors for US staging in comparison to other US signs. In addition, we found significant associations between conservative treatment or surgery and hypokinesis (*p* = 0.0326), akinesis (*p* = 0.0326), free fluid (*p* = 0.0013) and prominence of valvulae conniventes (*p* = 0.011). Free fluid in particular was significantly less present in patients that were conservatively treated (*p* = 0.040). We conclude that the US staging of SBO may be crucial, with a valuable role in the initial diagnosis and staging of the pathology, saving time and reducing total radiation exposure to the patient.

## 1. Introduction

Small bowel obstruction (SBO) is a common emergency department (ED) diagnosis; SBO may be functional, due to bowel wall or splanchnic nerve dysfunction, or mechanical. Mechanical SBO may be due to a luminal, mural or extra-mural mechanical barrier and may be proximal (high SBO) or distal (low SBO). Mechanical SBO occurs due to an impedance in the normal flow of intestinal contents with a partial or complete blockage of the small intestine. Partial obstruction allows some liquid contents and gas to pass through the point of obstruction, whereas complete obstruction impedes passage of all bowel contents. For these reasons, it often presents as a constellation of symptoms, and a diagnosis based on clinical presentation alone may not be reliable. Because SBO is a dynamic and ever-changing process that can resolve or worsen, it is staged into simple, decompensated and complicated. Failure to arrive at a timely diagnosis in the early stage of disease is an important cause of morbidity and mortality, resulting in significant complications such as strangulation and bowel necrosis [1,2,3,4]. The dilemma that surgeons and radiologists face when confronted with a possible SBO lies in confirming and staging the pathology. Imaging plays a significant role in making the diagnosis of SBO, as history and physical examination are unreliable [5]. In these scenarios, multimodality imaging (X-rays, ultrasound, CT and MRI) has been proposed to confirm, stage and define the cause of SBO [6,7,8,9]. CT represents the gold-standard imaging modality in the evaluation of SBO, answering to all diagnostic key points; in fact, it can confirm the pathology, determine the cause and level of mechanical obstruction and stage SBO, defining the presence or the absence of parietal damage. Recent studies demonstrated that ultrasound (US) and bedside point-of-care ultrasound (POCUS) have a reasonably high accuracy in diagnosing small bowel obstruction compared with CT scan, representing a rapid diagnostic modality to diagnose SBO, determining the presence or absence of pathology and substantially decreasing the time to diagnosis [10,11,12,13,14,15,16,17,18]. Ultrasound diagnosis of SBO can recognize the disease in an early stage, reduce time to diagnosis, limit patient radiation exposure and speed up the surgical or conservative management of SBO. Moreover, because stable patients with an ultrasound diagnosis of simple small bowel obstruction can be expeditiously admitted to the hospital in the surgical department, avoiding the need for a CT scan in the ED settings [11], decreasing health care ED costs. The purpose of this study was to evaluate the association and prevalence of sonographic morphological and functional signs that may be helpful in differentiating simple and advanced SBO (decompensated and complicated).

## 2. Materials and Methods

### 2.1. Subjects and Study Protocol

We performed a retrospective, single-center cohort study. We analyzed a convenience sample of adult ED patients presenting at our institution between January 2019 and December 2019 with an ultrasound diagnosis of SBO. The inclusion criteria was a positive ultrasound (US) diagnosis of SBO confirmed at computed tomography examination, on the discharge diagnosis for conservative treatment for SBO and/or on surgical and histological reports. Data were collected by reviewing the electronic medical record. Patients were eligible for enrollment if they were at least 18 years old, had not yet undergone imaging and presented with symptoms concerning for possible SBO. Potential subjects were identified by direct clinical contact by a member of the study team. US exams were carried out by a radiologist resident (G.M., one year of experience in emergency abdominal ultrasound) and alternately by two senior radiologists (S.T. and C.L., with >10 years of experience in emergency abdominal ultrasound).

All procedures performed in studies involving human participants were in accordance with the ethical standards (10297, 12/7/2019) of the institutional and/or national research committee and with the 1964 Helsinki declaration and its later amendments or comparable ethical standards.

### 2.2. Ultrasound Technique

Abdominal sonography was performed using a 2.5–3.5 MHz transducer in order to have a general overview of the abdomen using a curvilinear transducer at an imaging depth of 12–18 cm; a systematic evaluation of the entire abdomen was conducted. Extraintestinal causes of abdominal pain were first excluded and comorbidities were annotated in the report. Small bowel loops were searched in the central region of the abdomen and in the pelvis. Video clips of peristalsis and still images with appropriate measurements of the bowel loop diameter were saved. In addition, a focused examination with a linear probe (7–12 MHz) was performed when patients referred a focal point of tenderness. Interference by gas echoes from distended bowel was avoided by scanning the distended abdomen using oblique or coronal planes, or gentle pression through moving the transducer slowly over the abdomen (graded compression) was applied to squeeze the air away from the region of interest.

### 2.3. Ultrasound Diagnostic and Staging Criteria

US criteria for the diagnosis of SBO were related to morphological and functional findings [1] and divided into diagnostic criteria and staging criteria. Diagnostic criteria included the presence of dilated loops and abnormal peristalsis: small bowel dilatation was defined as bowel diameter 25 mm measured from outer wall to outer wall. Peristalsis was defined as hyperkinetic, decreased or absent. Ineffective peristalsis characterized by abnormal “back and forth” movements due to shuttling or swirling movements on intraluminal bowel contents was defined as decreased.

### 2.4. Staging Criteria Included Extraluminal Free Fluid, Parietal and Villi Alterations

The presence of free peritoneal fluid was considered positive if anechoic, extraluminal collections were visualized between bowel loops. Because there is no clear cut-off for the amount of free fluid, it is considered important to identify its presence or absence, assuming that the presence or reabsorption of free fluid between bowel loops is correlated with the worsening or resolution of the obstruction [15]. The parietal bowel’s alterations were related to the visualization of morphological changes of the bowel wall and were related to the thickness and stratified appearance of bowel wall. Wall thickness was defined as normal (1–3 mm), increased (>3 mm) or reduced (<1 mm) [18,19]. Parietal stratification was defined as two (double halo sign) or three (the target sign) concentric and symmetric layers of alternating echogenicity recognized on the thickened bowel wall. The circular folds (valves of Kerckring, plicae circulares or valvulae conniventes) are large valvular flaps projecting into the lumen of the small bowel. In case of bowel edema, the “plicae circulares” project into the bowel lumen, resulting in a “keyboard sign” [20], the prominence of valvulae conniventes was stated as present or absent. Ancillary findings reported were the evidence of groups of bowel loops with severe differences in diameter or kinesis, named bowel jump. Radiologists used a checklist data collection form to ensure all morphological and functional signs were obtained.

### 2.5. Statistical Analysis

Data were collected using standardized data collection forms. Data are presented as numbers and percentages for categorical variables, and continuous data are expressed as the mean ± standard deviation (SD) unless specified otherwise. Statistical analysis was performed by a senior biostatistician (N.S.).

The univariate and multivariate linear correlation analysis between US status and complications, such as free fluid between bowel loops, thickened or thinned walls, and the prominence of valvulae conniventes was performed, where the test on Pearson’s linear correlation coefficient R was performed with a Student’s *t*-test, under null hypothesis of Pearson’s linear correlation coefficient R = 0. 

In this step, for dichotomous variables, we assigned: dilated bowel wall: 1 = if > 2.5 cm and 0 if ≤ 2.5 cm; free fluid: yes = 1 and no = 0; thickened walls: yes = 1 and no = 0; thinned walls: yes = 1 and no = 0; prominence of valvulae conniventes: yes = 1 and no = 0; bowel jump diameter: yes = 1 and NE = 0 (not evaluable); and bowel jump kinesis: yes = 1 and NE = 0.

For the non-dichotomous variables, we assumed: Staging US: simple = 1, decompensated = 2, complicated = 3, hyperkinesis = 1, hypokinesis = 2, akinesis = 3; Therapy: conservative (conservative) = 1, emergency surgery (surgery) = 2, failure of medical treatment and surgery (conservative + surgery) = 3.

The multiple comparison chi-square test was used to define significant associations or dependence among therapy groups: conservative, surgery and conservative + surgery and US signs identified such as hyperkinesis, hypokinesis, akinesis, free fluid, thickened walls, thinned walls, prominence of valvulae conniventes, bowel jump kinesis, bowel jump diameter and dilated bowel. In this case, if the chi-square test was positive (*p*-value less than 0.05), then the post-hoc Z-test was performed.

The multiple comparison Cochran’s Q test was used to compare the differences among percentages under the consideration of the null hypothesis that there were no differences between the variables. When the Cochran’s Q test was positive (*p*-value < 0.05), then a minimum required difference for a significant difference between two proportions was calculated using the minimum required differences method with the Bonferroni *p*-value corrected for multiple comparisons.

All tests with a *p*-value < 0.05 were considered significant. The statistical analysis was performed in the Matlab statistical toolbox version 2008 (MathWorks, Natick, MA, USA) for Windows (32-bit).

## 3. Results

Of 63 consecutive patients, 28.57% (18/63) were males and 71.43% were females (45/63), with ages at operation in the range of 33–87, with a mean of 68.69 years old and standard deviation of 17.72 years. Our participants were mostly women (45\63): by review of the electronic medical record, 20/45 underwent a cesarean section, 10/45 had a history of endometriosis and 15/45 had previous abdomen and/or pelvic surgery. All men had had previous surgery. In Table 1, we report the characteristics of the 63 participants in this study.

In Table 2 we report the results of univariate and multivariate linear correlation analysis, considering US staging as dependent variable and as independent variables complications such as the presence of free fluid, abnormal peristalsis, as well as parietal and valvulae conniventes alterations.

Table 2 shows by univariate analysis a positive linear correlation between US staging and free fluid (*R* = 0.76, *p* < 0.0001), thickened walls (*R* = 0.42, *p* = 0.0005) and the prominence of valvulae conniventes (*R* = 0.25, *p* = 0.498). Instead, in multivariate analysis, a positive predictor of US staging was the presence of free fluid, while thinned walls (*R_partial_* = −0.28; *p* = 0.0398) and bowel jump (*R_partial_* = −0.29; *p* = 0.0320) were negative predictors. By these results, we observed a significant correlation in both multivariate and univariate analyses with the presence of free fluid. In other words, the more advanced the stage of SBO, the more probable the presence of free fluid between the bowel loops. 

In univariate analysis, we found that the presence of thickened walls or prominence of valvulae conniventes implicated a more advanced US SBO stage.

Multivariate analysis indicated that parietal stratification and bowel jump kinesis were negative predictors for US staging, in comparison to other variables. In other words, the absence of parietal stratification and bowel jump kinesis were predictors of a simple SBO, considering all variables simultaneously. We underline that the dilated bowel variable was not included in the multiple regression model, because in this case the regression equation was not soluble, and therefore we considered it only in the univariate analysis.

From the multiple-comparison Cochran’s Q test, we observed that there were significant differences (*p* < 0.001) between the variables hyperkinesis, hypokinesis, akinesis, free fluid, thickened walls, thinned walls, the prominence of valvulae conniventes, bowel jump kinesis, bowel jump diameter and dilated bowel. The post-hoc test indicated that the most significant complications present were: dilated bowel (100%, *p* < 0.05), hypokinesis (90.46%, *p* < 0.05), thickened walls (82.54%, *p* < 0.05) and free fluid (74.60%, *p* < 0.05).

Finally, we considered three groups according to clinical and surgery therapy scale: conservative, surgery and conservative + surgery, and reported for every group the frequency of US findings for all independent variables considered in Cochran’s Q test, as shown in Table 3. 

From Table 3, a significant association between therapy group and hypokinesis (*p* = 0.0326), akinesis (*p* = 0.0326), free fluid (*p* = 0.0013) and the prominence of valvulae conniventes (*p* = 0.011) can be observed. Particularly, the post-hoc Z-test was significant only for free fluid, and it showed that free fluid was significantly less present in the conservative group (*p* = 0.040).

## 4. Discussion

**Figure 1:**
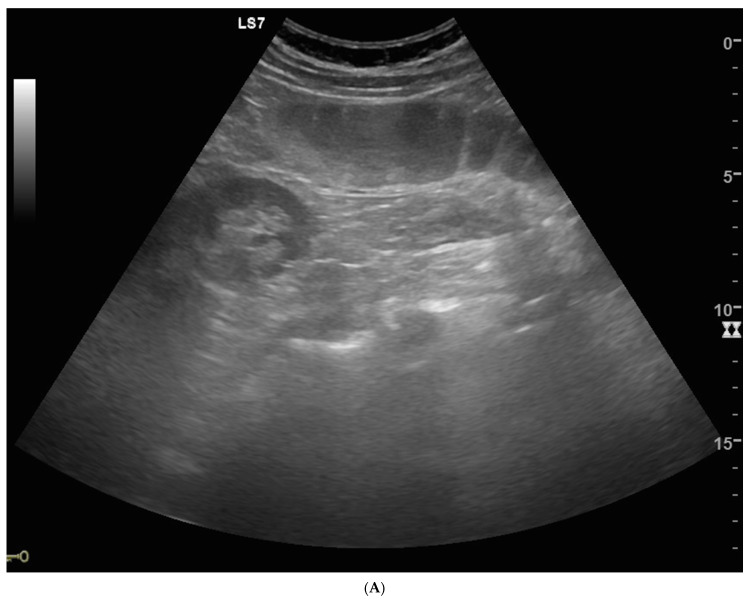

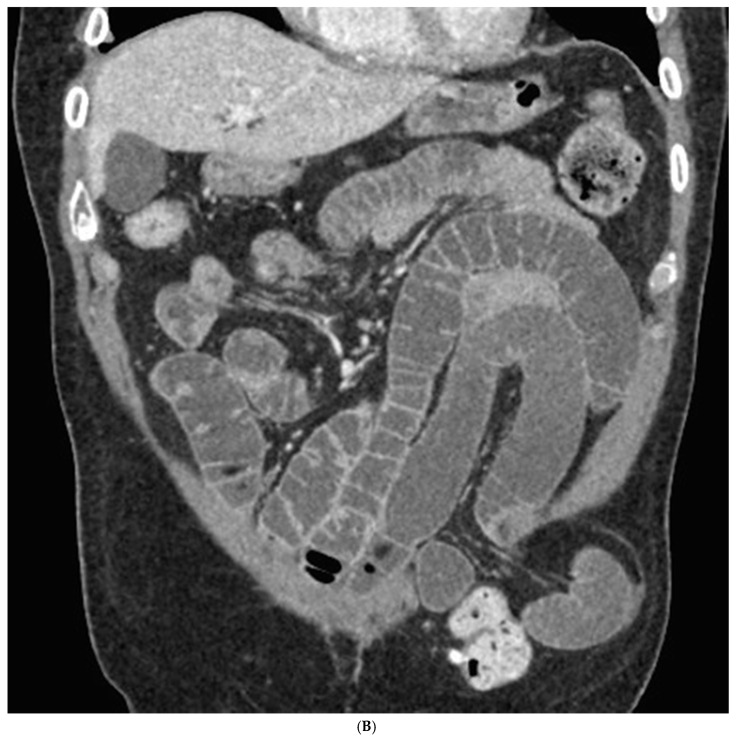
Simple SBO. (**A**) Dilated and fluid-filled bowel loops with decreased peristalsis; valvulae conniventes are clearly visible but not thickened. No free fluid was detected in the abdominal cavity or between bowel loops. (**B**) CT with IV contrast, coronal MPR, confirmed a simple SBO diagnosis in caused by a left spigelian hernia.

**Figure 2:**
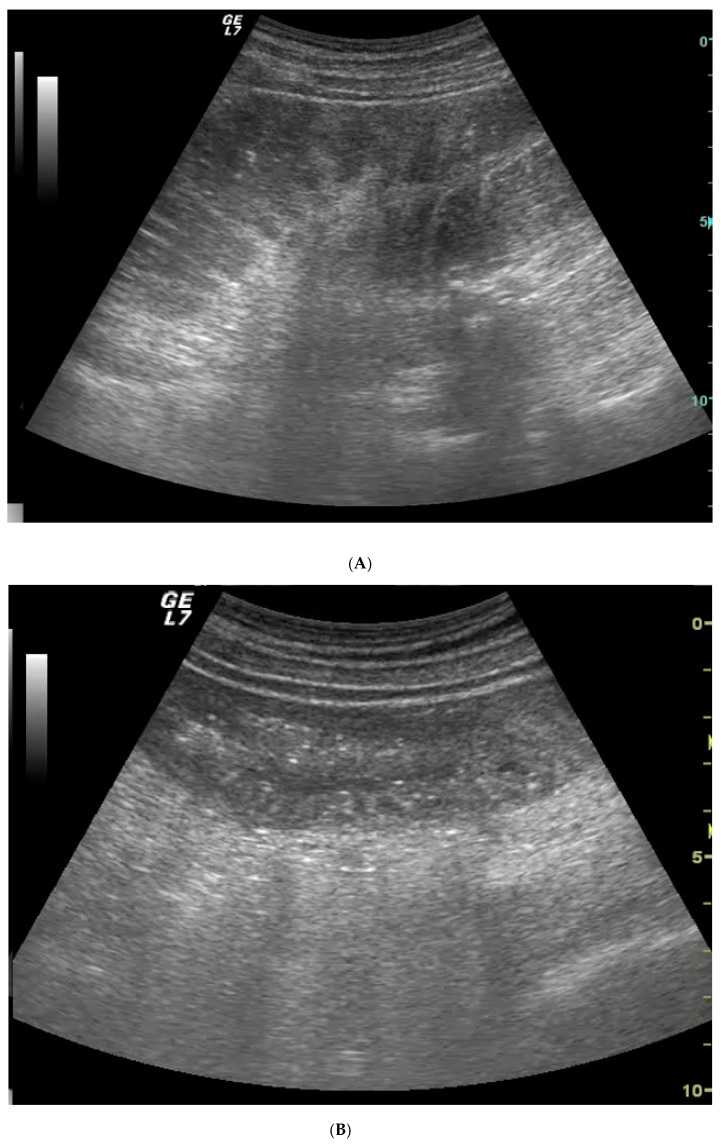
Simple SBO at the time of diagnosis (**A**) and after positioning of nasogastric tube insertion (**B**). (**A**) Dilated and fluid-filled bowel loops; valvulae conniventes are clearly visible but not thickened. (**B**) After nasointestinal decompression, bowel loops are not dilated. Peristalsis is recovered.

**Figure 3:**
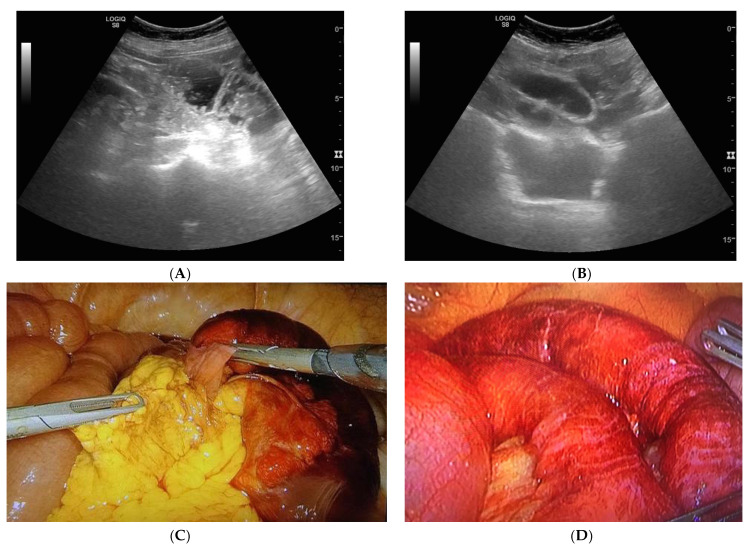
Decompensated SBO. (**A**,**B**) Free fluid between bowel loops, the bowel is dilated, hypo/akinetic, and valvulae conniventes are clearly visible and thickened. (**C**) Laparoscopic view of an SBO due to an epiploic bridle with no evident venous distress of the dilated intestinal loop. (**D**) Laparoscopic view of the same intestinal loop after the section of the bridle; the bowel loop recovered a normal appearance and peristalsis with slight hyperemia.

**Figure 4:**
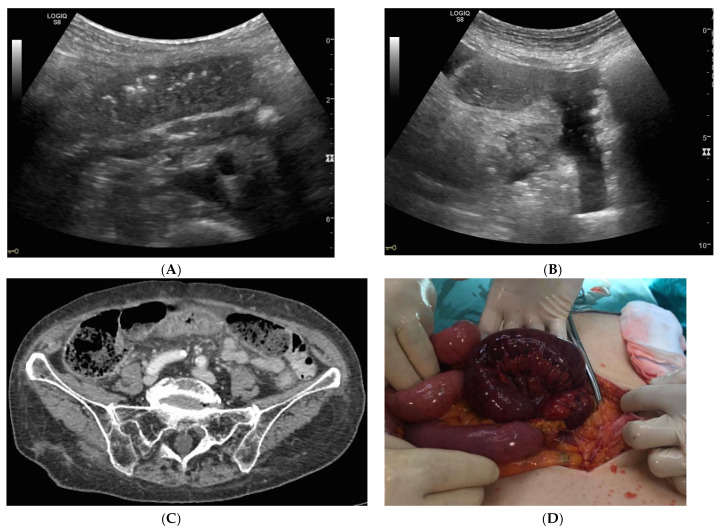
Complicated SBO. (**A**,**B**) Bowel loops are dilated, akinetic, with parietal and valvulae conniventes thickening and parietal stratification. There is free fluid between bowel loops. (**C**) CT with IV contrast axial image. Bowel loop with thickened and stratified wall with hypointense submucosa (image A). In the left quadrant, a dilated bowel loop with a thin layer and feces sign is visible (image B). (**D**) Laparotomic view of a dilated intestinal loop in necrosis.

The US diagnostic criteria of bowel obstruction are the presence of dilated bowel loops (>2.5 cm) and abnormal peristalsis. Because the indications for and the timing of surgical intervention for SBO have changed over the past several decades, there is a widespread assumption that the majority of patients with simple SBO can be conservatively managed with nasogastric tube insertion, bowel rest and IV fluids, and do not require operative intervention [4,10,11]. Because SBO is a dynamic pathology that can resolve or evolve, other signs have been advocated for in the staging of SBO,—signs that determine the stage of disease based on bowel parietal damage. In this scenario, staging SBO is crucial. Initially, the obstacle to the progression of intestinal contents and fluids determines dilatation and fluid-filled bowel loops. Bowel loops proximal to the point of obstruction in the initial phase may be hyperperistaltic, and valvulae conniventes may be clearly visible but not thickened because the bowel is contracting more to try to overcome the obstruction. In ultrasound, bowel loops appear dilated (>2.5 cm) with no parietal thickening and present abnormal peristalsis (simple SBO) (Figure 1A,B and Figure 2A,B). The movement of the mechanically obstructed bowel will decrease with the persistence of obstruction, and with the increase of endoluminal pressure that determines the bowel’s inability to resorb liquids: bowel layers act as a sponge, determining the passage of fluid in the peritoneal cavity [15]. In ultrasound imaging, the first sign that indicates the worsening of the pathology is the presence of free fluid between bowel loops; the bowel is dilated, hypo or akinetic, but valvulae conniventes are clearly visible and thickened (decompensated SBO) (Figure 3A–D). As time passes, vascular damage progresses, and in this phase, bowel layers are thickened due to vascular parietal damage [18]. Additionally, in this phase the bowel loops are seen in the ultrasound as dilated, akinetic, with parietal and valvulae conniventes thickening, parietal stratification and free fluid between bowel loops (complicated SBO) (Figure 4). Sonographic peristalsis evaluation and grading is operator-dependent and there is no consensus statement in measurements. Despite a lack of consensus, many radiological and radiological studies performed by residents and/or specialists have demonstrated that adequate training supports the correct definition and grading of abnormal peristalsis [3,4]. The results of this study demonstrate that ultrasound findings reflect the pathological evolution of small bowel obstruction. 

## 5. Conclusions

Our study highlights and supports the utility of US examination for the diagnosis and management of patients with suspected SBO and introducing an emerging role for the US staging of SBO. The results of our study demonstrate that the dilation of the loops and the alteration of kinetics have a sensitivity (94%) and a specificity (96%) in the diagnosis of SBO [12], and that, in the staging of SBO, the presence of liquid and thick walls are the criteria for high sensitivity and diagnostic specificity for staging a decompensated or complicated SBO. 

Further prospective studies on large case series are required.

## Figures and Tables

**Table 1:** Characteristics of the 63 study participants.

Parameters	Mean/Percentage
Gender	
Male	28.57% (18/63)
Female	71.43% (45/63)
Age (years)	68.69 ± 17.72
Therapy	
Conservative	42.86% (27/63)
Surgery	49.21% (31/63)
Conservative + Surgery	7.94% (5/63)
US status	100%
Simple	26.98% (17/63)
Decompensated	22.22% (14/63)
Complicated	50.79% (32/63)
Free fluid (yes)	74.60% (47/63)
Thickened walls (yes)	82.54% (52/63)
Thinned walls (yes)	44.44% (28/63)
Prominence of valvulae conniventes (yes)	42.86% (27/63)
Dilated bowel (>2.5 cm)	100%
Peristalsis	
Hyperkinesis	0%
Hypokinesis	90.48% (57/63)
Akinesis	9.52% (6/63)
Bowel jump diameter	
Yes	55.56% (35/63)
Not evaluable (NE)	44.44% (28/63)
Bowel jump kinesis	
Yes	36.51% (23/63)
NE	63.49% (40/63)

**Table 2:** Linear correlation analysis between dependent variable ultrasound (US) staging and independent variables.

Linear Correlation Analysis	Univariate Analysis *R* (*p*-Value)	Multivariate Analysis *R_partial_*; *p*-Value
		Multiple linear correlation coefficient = 0.82
US staging/dilated bowel #	0.00 (1.00)	—
US staging/peristalsis	0.03 (0.83)	*R_partial_* = 0.19; *p*-value = 0.17
US staging/free fluid	0.76 (<0.0001) *	*R_partial_* = 0.75; *p*-value < 0.0001 *
US staging/thickened walls	0.42 (0.0005) *	*R_partial_* = 0.11; *p*-value = 0.40
US staging/thinned walls	0.09 (0.49)	*R_partial_* = −0.26; *p*-value = 0.048 *
US staging/prominence of valvulae conniventes	0.25 (0.0498) *	*R_partial_* = 0.10; *p*-value = 0.44
US staging/bowel jump diameter	−0.01 (0.92)	*R_partial_* = 0.19; *p*-value = 0.15
US staging/bowel jump kinesis	−0.14 (0.29)	*R_partial_* = −0.27; *p*-value = 0.040 *

* *p* < 0.05; R: Pearson’s linear correlation coefficient; *R_partial_*: the partial correlation coefficient is the coefficient of correlation of the variable with the dependent variable, adjusted for the effect of the other variables in the mode; # dilated bowel variable was used only in univariate analysis.

**Table 3:** Multi-comparison chi-square tests between therapy group and US signs.

US Sign/Groups	Conservative N. 27	Surgery N. 31	Conservative + Surgery N. 5	Statistical Analysis *p*-Value (Test Type)
Dilated bowel	100%	100%	100%	*p* = 1.00 (C)
Hyperkinesis	0.0%	0.0%	0.0%	*p* = 1.00 (C)
Hypokinesis	100%	80.65% (25/31)	100%	*p* = 0.0326 * (C), NS
Akinesis	0.0%	19.4% (6/31)	0.0%	*p* = 0.0326 * (C), NS
Free fluid	51.9% (14/27)	93.6% (29/31)	80% (4/5)	*p* = 0.0013 * (C), NS Conservative ***, *p* = 0.040 (Z)
Thickened walls	70.4% (19/27)	90.3% (28/31)	100%	*p* = 0.077 (C)
Thinned walls	40.7% (11/27)	45.2% (14/31)	60% (3/5)	*p* = 0.72 (C)
Prominence of valvulae conniventes	22.2% (6/27)	61.3% (19/31)	40% (2/5)	*p* = 0.011 * (C), NS
Bowel jump diameter	55.6% (15/27)	51.5% (16/31)	80% (4/5)	*p* =0.50 (C)
Bowel jump kinesis	37.0% (10/27)	35.5% (11/31)	40% (2/5)	*p* = 0.98 (C)

* *p* < 0.05; ** most frequent; *** least frequent; C: multiple-comparison χ^2^ test; Z: post-hoc Z-test; patients generally had more than one US sign; NS = non-significant post-hoc Z-test.
